# Prefrontal Cortex Modulation during Anticipation of Working Memory Demands as Revealed by Magnetoencephalography

**DOI:** 10.1155/2010/840416

**Published:** 2010-06-28

**Authors:** Mario Altamura, Terry E. Goldberg, Brita Elvevåg, Tom Holroyd, Frederick W. Carver, Daniel R. Weinberger, Richard Coppola

**Affiliations:** ^1^Clinical Brain Disorder Branch, NIMH, Bldg. 10, Rm. 4S235, Bethesda, MD 20892, USA; ^2^Psychiatry Unit, Department of Medical Sciences, University of Foggia, Foggia 71100, Italy; ^3^MEG Core Facility, National Institutes of Health, Bldg. 10, Rm. B1D65, Bethesda, MD 20982, USA

## Abstract

During the anticipation of task demands frontal control is involved in the assembly of stimulus-response mappings based on current goals. It is not clear whether prefrontal modulations occur in higher-order cortical regions, likely reflecting cognitive anticipation processes. The goal of this paper was to investigate prefrontal modulation during anticipation of upcoming working memory demands as revealed by magnetoencephalography (MEG). Twenty healthy volunteers underwent MEG while they performed a variation of the Sternberg Working Memory (WM) task. Beta band (14–30 Hz) SAM (Synthetic Aperture Magnetometry) analysis was performed. During the preparatory periods there was an increase in beta power (event-related synchronization) in dorsolateral prefrontal cortex (DLPFC) bilaterally, left inferior prefrontal gyrus, left parietal, and temporal areas. Our results provide support for the hypothesis that, during preparatory states, the prefrontal cortex is important for biasing higher order brain regions that are going to be engaged in the upcoming task.

## 1. Introduction

While performing a task, attention is allocated to each successive stage of task processing, including the anticipation of incoming events. “Anticipation” refers to an expectation in which the various demands of an upcoming task are prospectively configured. Data from studies in primates suggests that the ability to anticipate forthcoming events depends on the activity of the frontal lobes [1]. Neuroimaging studies have examined anticipation in a variety of controlled paradigms in which a predesignated response is elicited by an upcoming imperative stimulus [2]. These studies suggest that the function of the prefrontal cortex (PFC) is the assembly on an *ad hoc* basis of stimulus–response mappings based on current goals. Few studies, however, have examined anticipation during complex task preparation [3–5]. Crucially, it is not clear whether prefrontal modulations occur in higher order cortical regions, likely reflecting cognitive anticipation processes. 

 It has been postulated that PFC may anticipate the imminent stimuli by exerting top-down control in a manner similar to that which occurs during the performance of a working memory (WM) task [6]. During preparatory states [7] and performance of a WM task [8] there is often a substantial reorganization of brain rhythms. Several neurophysiological studies, however, have reported different patterns of electroencephalographic activities between preparatory periods and performance of WM tasks [9–11]. The principal interest in the current study was the anticipation-related activity preceding working memory (WM) demands and the prediction that the patterns of electroencephalographic activities would differ between the preparatory periods and the performance of WM trials. Changes in brain activity have been measured as relative changes of EEG power in which a relative decrease in power is called event-related desynchronization (ERD) and relative increase in power is called event-related synchronization (ERS). It has been suggested that the cortex is deactivated before memory tasks (and other complex cognitive processes). Particularly, it has been hypothesized that memory performance is enhanced if the cortex is deactivated before a task is performed and that the initial inhibition of the cortex preceding cognitive performance may reflect inhibitory top-down control [12]. Beta frequency measures are appropriate for revealing manifestation of inhibitory top-down control during anticipation of upcoming working memory demands. Increase in beta power (ERS) after movement execution (so-called “beta rebound”) has been interpreted as a sign of functional inhibition of sensorimotor cortical areas in motor tasks [13, 14]. Some studies have suggested that beta rhythm ERS responses may reflect the “active inhibition” of the motor cortices when a motor activity is repressed [15, 16]. This interpretation is supported by the findings of beta synchronization (ERS) over the sensorimotor areas related to the withholding of movement in NoGo trials [17] and in the frontal electrodes during the recognition stage of a memory scanning task associated with the active inhibition of responding [8]. Studies in nonhuman primates have reported specific patterns of EEG synchronized activity in the beta frequency band in the PFC when the monkey anticipates the presentation of a visual stimulus [18, 19]. In humans some studies have suggested that phase synchronization in a fronto-parietal network with frequencies in the beta band may play an important role in top-down control during the anticipation of visuomotor reaction time tasks [20]. An increase in EEG beta power over frontal areas before presentation of an arithmetic task has been reported in humans [21]. Based on these previous findings we hypothesized an enhancement of energy specifically in the beta frequency during the preparatory periods before the presentation of WM trials. There have been numerous reports on the modulation of beta band (14–30 Hz) activity in humans performing working memory tasks [22–25]. Thus, it has been suggested that beta oscillations may reflect cognitive processing in addition to the activity of the motor cortices seen in earlier work [26]. While an increase in EEG beta power over frontal areas before presentation of cognitive task has been reported [21], beta ERD have been observed in the frontal electrodes during the different stages of a working memory task [8]. A number of studies have previously observed a characteristic prestimulus synchronization followed by a powerful poststimulus desynchronization in the beta frequency during a nonmotoric task [27, 28]. Based on these findings we hypothesized that power in the beta band may vary with anticipation and memory, with an enhancement of energy in the beta frequency during the preparatory periods before the presentation of WM task followed by a significant decrease in beta power during WM task related processes. We also hypothesized that anticipation would be associated with power changes over higher order associative cortical areas pertinent to the anticipated WM trials. Comparing the ERS/ERD patterns emerging during the preparatory periods with the ERS/ERD changes occurring during the performance of WM task would afford us the unique opportunity to test the hypothesis that WM performance and preparation elicit different patterns of activity. 

## 2. Methods

### 2.1. Study Participants

Twenty right-handed healthy volunteers (12 males; mean age 27.6 years) participated. All were screened against medical, neurological, and psychiatric illnesses, and for use of prescription medications. The study was conducted according to the guidelines of the internal review board at the National Institute of Mental Health. All participants signed consent forms after the procedure had been explained to them.

### 2.2. Behavioral Procedures

**Figure 1:**
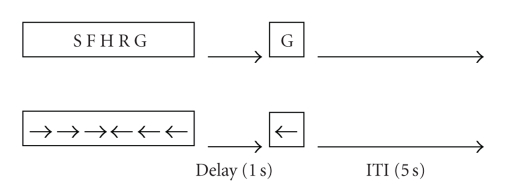
Trial sequence in the working memory (letters) and control (arrows) conditions. Three consecutive working memory trials alternated with three consecutive control trials. ITI = intertrial interval.

Participants were seated and instructed to keep as still as possible during the experiment. A modified version of the Sternberg memory task [29] was employed which consisted of: (i) the encoding phase (2 s) during which a set of 5 letters was presented, followed by (ii) the delay period (1 s) during which a fixation point appeared in the centre of the screen, and finally (iii) the response period (2 s) during which a single letter probe was presented. Participants were instructed to read the letters from left to right and to press a button (held in the dominant right hand) as quickly and accurately as possible according to whether the probe had been present (positive probe) or absent (negative probe) in the preceding string. During the response period participants indicated with a right button press if the letter was a member of the set they had viewed at encoding and with a left button press if it was not. There was an equal proportion of negative to positive probes. The letters (all consonants) in each trial were selected in a pseudorandom manner with the restriction that there were no stimulus overlaps between the strings (or probes) in consecutive trials. The control task followed the identical structure with the only difference being that letters were substituted with a set of 4 horizontal left and right arrows pointing towards the center of the screen, which were presented followed by a single arrow pointing either to the left or to the right, to a which a button in the corresponding direction was to be pressed. Each WM and control trial lasted 5 s followed by an intertrial interval (ITI) of 5 s of no stimuli (blank screen). There were 3 trials in each consecutive block, thus totaling 30 sec. The experimental and control tasks were alternated each block. Therefore, three consecutive working memory trials were alternated with three consecutive control trials. There were 24 blocks of trials (each block has three trials), thus resulting in 72 trials (36 working memory trials + 36 control trials). Since participants knew in advance what type of task would be presented on the next trial, anticipation was likely high. For current purposes, preparatory periods were operationalized as the 5 s intertrial intervals starting from the moment the response period ended until the onset of the encoding phase of the next trial (Figure 1).

### 2.3. MEG Acquisition Procedures

Data were collected in a magnetically shielded room (Vacuumschmelze, Germany) using a CTF 275 MEG system (CTF Systems Inc., Coquitlam BC, Canada) composed of a whole-head array of 275 radial 1st order gradiometer/SQUID channels with the participants positioned 15 degrees from vertical. The head position was determined with localization coils at the nasion and preauricular fiducial points. Before and after each MEG recording these points were localized by detecting the magnetic signals transmitted by the three coils. The two localizations were compared to check the head movement of each participant during the experimental session. In all cases head movement did not exceed 0.5 cm. MRIs were acquired on a 1.5 Tesla GE scanner with 124 sagittal slices (thickness 1.5 mm). In order to coregister the MRI and MEG data three fiducial markers were placed on the MRI at the same locations used during MEG data acquisition.

### 2.4. Data Analysis

MEG signals were sampled at a rate of 600 Hz and then high-pass filtered at 0.61 Hz. Markers were set in the data for the presentation of the memory sets and the probes, as well as for each participant's response. Synthetic aperture magnetometry (SAM) was used to reconstruct whole brain volumes in the beta frequency band (14–30 Hz). SAM estimates the volume of activation using a spatial filtering approach which optimizes the discrimination between the signals that arrive from a target voxel from those originating from other possible simultaneous active sources. The sources are linear estimations of the signals described by covariance matrices over discrete time windows relative to an event (see below). We derived brain shape models by first stripping the skull from the structural data [30]. The brain shape was used for forward modeling of the MEG signals. SAM analyses can be used in a dual state design, with epochs designated as active or control, and the resulting brain volumes representing the relative power difference between the two states. SAM analysis estimates the source power for each voxel in the brain, using a beamformer. Because of the way beamformers work, the raw source power estimate increases with depth, and so must be normalized. We used a dual-state imaging, in which the normalization is done using real brain noise, a so-called *control* state. In this case the state being normalized is called the *active* state. SAM creates an optimal spatial filter from the covariance between the active state and the control state to calculate a 3d source image comparing the source strength for the specified time windows of the two states. A calculation of source power was performed for 7.5 mm^3^ voxels throughout the brain volume. The amplitude was obtained by computing a *pseudo-F *ratio between the power in the active and the control state. In the case of ERS, the *pseudo*-*F* value is derived from the formula *A*/*C* − 1, where *A* is the source power in the active state and *C* is the source power in the control state. For ERD, the formula is −((*C*/*A*) − 1). By definition the *pseudo-F* is the ratio of variance. With respect to a multiple-trial MEG study, SAM determines the variance of source of power for the active state, and variance of source of power for the control state. That is, there are independent estimates of power for each trial at each voxel. We used the term *pseudo*-*F* because the ratio of source power over all active trials to source power over all control trials “resembles” the *F-ratio*. However it does not compute the *F* distribution and cannot be used to estimate a probability value. The raw Active/Control is not a real *F* statistic (the degrees of freedom are not taken into account). A SAM run consists of computing a *covariance matrix* from the MEG data using SAMcov, and then using that covariance matrix to estimate source power inside the brain using SAMsrc (http://kurage.nimh.nih.gov/meglab). Therefore, the SAM volumes, for each participant, contain a power ratio values. 

 To reveal beta power reactivity to the anticipatory periods the first trials from both experimental and control task were excluded. For analysis of brain activity, only trials on which responses were correct were included. To define the spatiotemporal sequence of those cerebral regions active during different phases of the experiment, the SAM analyses were performed on the entire active epoch (preparatory periods, encoding, delay and retrieval). Trials were segmented using a sliding 500 ms time window with 100 ms increments and compared to the corresponding time window in the control task. 

**Figure 2:**
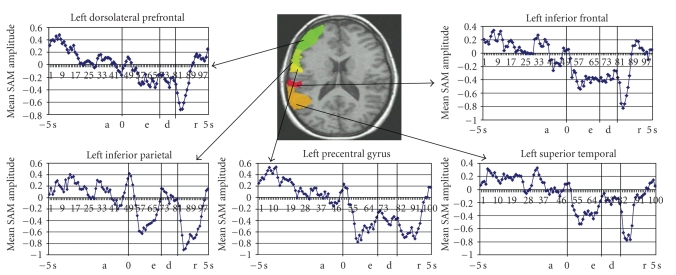
ROI templates drawn on a representative participant's MRI scan along with the average time courses from each of the left hemisphere ROIs. a: anticipatory period; e: encoding period; d: delay; r: response period.

**Figure 3:**
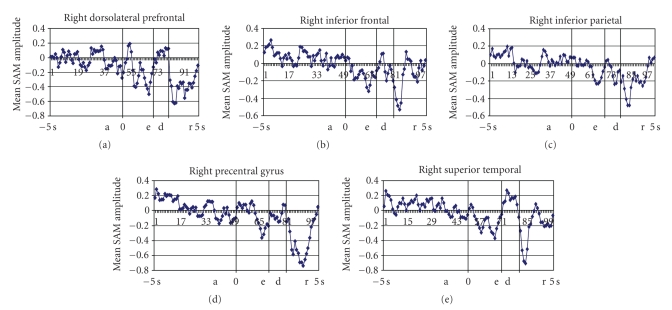
Time courses from each of the right hemisphere ROIs. a: anticipatory period; e: encoding period; d: delay; r: response period.

 The SAM volumes were then normalized to Z-scores. Z-scoring is done by taking the mean over the volume, subtracting it, and then dividing by the standard deviation over the same volume. (http://kurage.nimh.nih.gov/meglab; 3dNormalize reads the.svl file and scales the values to have a standard deviation of 1, producing an AFNI BRIK. With the −Z option, it also removes the mean, producing Z -scores). Then, we get statistic computing *t-*test at each time window over the course of experiment to test the hypothesis that the active state had significantly more or less power than the control state. The random permutation analyses were performed for each time window to take care of the problem that the voxels are not computed independently. With permutation testing the statistic (*t*-test) on the observed data is calculated for all combinations of the data that are possible under the null hypothesis. Under the null hypothesis in the present experiment there is no difference between the active and control conditions. Random permutation analysis performed a *t*-test over and over with shuffled data (active and control conditions across participants) to build up a distribution of statistical values for each voxel. By doing this all possible *t*-values can be calculated which provides an exact distribution of the *t*-statistic for this data set. Then, one can evaluate the* t*-value obtained with the original data set and determine how many times that value was exceeded out of all the possible *t*-values. These values are used to calculate corrected *P* *-*values that more accurately reflect the pervoxel statistics. All reported effects in these analyses were reliable at the *P* < .01 level unless otherwise indicated. To analyze the group data in a common anatomical space the structural data and the SAM volumes were aligned into Talairach space using Analysis of Functional NeuroImages (AFNI) [30]. Functional data were further analyzed using a region of interest (ROI) approach. To examine MEG activity in specific cortical regions, separate regions of interest (ROIs) were created for each participant. Regions were defined on the basis of statistically significant activations revealed by combining all epochs including preparatory and working memory periods (encoding, delay, response). The defined ROIs were in close agreement with data in the literature for verbal working memory. The MRI scans were used to draw individualized ROI templates for each participant corresponding to a standardized coordinate frame from the Talairach and Brodmann atlases. In light of some individual anatomical variability the ROIs were adjusted to better correspond to the anatomical images of some participants in order to more accurately demarcate the intended brain regions. The analyses focused on five relevant ROI's per hemisphere. The prefrontal dorsolateral ROIs corresponded to Brodmann's areas 9 and 46; the inferior frontal ventrolateral ROI's corresponded to Brodmann's areas 44, 45 and 47; the premotor ROI's corresponded to Brodmann's area 6; the parietal ROI's corresponded to Brodmann's area 40; the temporal ROIs corresponded to Brodmann's area 22. Both hemispheres were examined because the right homologues of many cortical areas have been shown to be activated by the same type of cognitive processing as their left counterparts in verbal working memory task. These individualized ROI templates were used to interrogate the mean images series, which reflect statistical analyses (*t*-tests) preformed for each time window on the entire active epochs (preparatory periods, encoding, delay and retrieval). This was done separately for each participant and each ROI. The time course of the MEG signals over the course of experiment were extracted at each time point for each participant within each ROI and then averaged across participants ( Figures 2 and 3 ).

## 3. Results

### 3.1. Behavioral Data

 We report accuracy of responses and reaction time. The mean percentage of correct answers was 95 (SD 5.03). The mean percentage of correct answers in the first, second, and the third WM trials, respectively, were 91.5 (SD 5.6), 90.5 (SD 5.7), and 89.7 (SD 7.2). A repeated measures analysis of variance (ANOVA) using the mean percentage of correct answers for each WM trial as within-subject factors is not significant (*F*_2,38_ = 2.19, *P* = .12). Mean reaction time was 824 ms (SD 224). Mean reaction time in the first, second and third WM trials, respectively, were 815 ms (SD 212), 818 ms (SD 229) and 838 ms (SD 276). The analysis of repeated measures of ANOVA using the mean reaction times for each WM trial as within-subject factors is not significant (*F*_2,38_ = 0.265, *P* = .76). 

### 3.2. MEG Data

#### 3.2.1. Anticipation (Time Period During Intertrial Intervals)

**Figure 4:**
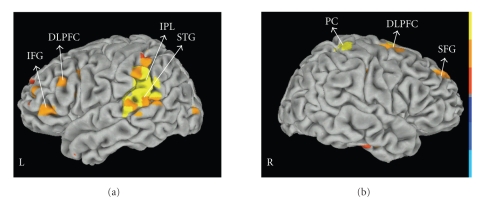
Group maps corresponding to 200 ms interval (from −1700 ms to −1500 ms) preceding the trials onset at beta frequency (14–30 Hz); *P* < .01 corrected; DLPFC: dorsolateral prefrontal cortex; IFG: Inferior frontal gyrus; IPL: inferior parietal lobe; PC: postcentral gyrus; SFG: superior frontal gyrus. Red color coding indicates task related power increase; blue color coding indicates task related power decrease. The figure shows data rendered onto a Talairach-space surface template.

Compared with the control condition the experimental condition displayed a beta power increase (ERS) in dorsolateral prefrontal cortex (DLPFC) bilaterally, left inferior prefrontal gyrus (BA 45/47), left parietal regions (BA 40), left superior temporal areas (BA 22), right superior frontal gyrus and right post central gyrus (*P* < .01 corrected) (Figure 4). The pattern of activation over time showed beta ERS (1700 ms before the onset of the letter sequence) in left DLPFC, inferior prefrontal gyrus and superior temporal area, followed (from −1500 to −1300 ms) by ERS over left DLPFC and left superior temporal regions. No voxels correlated with reaction time in any of the time windows. Visual inspection of the individual differences in the activation over DLPFC confirmed the presence of beta power increases in all but four participants. There was variability across the remaining participants in the temporal dynamics of beta ERS shifts in prefrontal sites. However, the increase of beta power in PFC peaked within 2000 ms preceding the onset of the working memory trials in all participants.

#### 3.2.2. ROIs Analyses

To examine interregional differences in the selected ROIs we performed a repeated measures ANOVA of SAM amplitude responses for the preparatory period (epochs centered on −2000 ms before the onset of WM trials). This analysis demonstrated that the selected cortical regions were differentially activated during the anticipation of the task (*F*_9,171_ = 4.46, *P* < .0001). The major effects of anticipation were in the left DLPFC and left superior temporal gyrus. 

#### 3.2.3. Correlation of Activation and Behavioral Performance

 We found no significant correlation for any regions between activation during preparatory periods and the RTs of the subsequent task performance.

#### 3.2.4. Encoding

**Figure 5:**
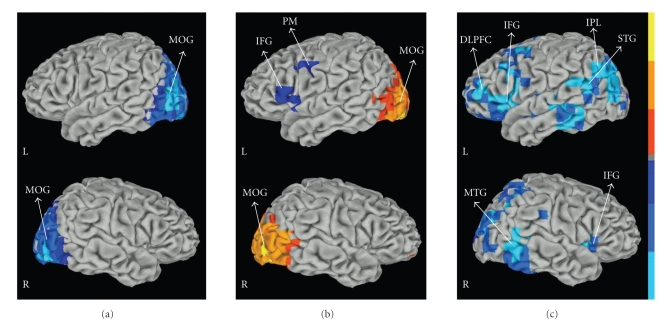
Group maps during the encoding, delay and retrieval epochs of the working memory task at beta frequency (14–30 Hz); *P* < .005 corrected. (a) The encoding epoch was associated with fairly symmetrical ERD in primary visual and visual association cortex. The map shows an epoch of 500 ms duration (from 200 ms to 700 ms after the memory set presentation). (b) The delay epoch was associated with beta ERS in bilateral visual areas and beta ERDs in left IFG and left premotor regions. The map shows an epoch of 400 ms duration (from 300 ms to 700 ms after the delay began). (c) The response period beta was associated with beta ERDs over a distributed network including left DLPFC, left IFG and premotor areas, right IFG, temporal and parietal regions. The map shows an epoch of 300 ms duration (from 0 ms to 300 ms after the presentation of the probe. DLPFC: dorsolateral prefrontal cortex; IFG: inferior frontal gyrus; PM: premotor area; IPL: inferior parietal lobe; STG: superior temporal gyrus; MTG: middle temporal gyrus; MOG: middle occipital gyrus. Red color coding indicates task related power increase; blue color coding indicates task related power decrease. The figure shows data rendered onto a Talairach-space surface template.

The encoding epoch was associated with fairly symmetrical ERD in primary visual areas (BA 17/18), and visual association cortex, including middle occipital gyrus (BA 19) and precuneus (BA 7) (*P* < .005 corrected) (Figure 5). The spatiotemporal sequence indicates that these regions were active as early as 200 ms after the memory set presentation and remained active during the entire encoding epoch. Beta ERD activities were elicited in the left DLPFC (BA 9) from 1300 to 1900 ms, left premotor areas (BA 6) from 1000 to 1900 ms and left superior temporal gyrus from 1000 to 1600 ms after working memory trial onset.

#### 3.2.5. Delay

The delay epoch was associated with beta ERSs in bilateral visual areas (BA 17; BA 18) in the occipital lobes including precuneus (BA 7) and middle occipital gyrus (BA 19). ERSs in visual area appeared as early as 200 ms after the delay period began and remained sustained during the entire delay (*P* < .001 corrected). Delay-related ERD was observed in left inferior frontal gyrus (BA 44/45), left premotor cortex (BA 6) and left DLPFC (left BA9) (*P* < .005 corrected). The spatiotemporal course showed that decrease in beta power was located (from 0 to 400 ms) in left inferior frontal gyrus and premotor areas (Figure 5). During the last 200 ms of the delay (from 800 to 1000 ms), the mean ERD was located in left DLPFC (BA 9) and left inferior frontal gyrus (BA 45/47).

#### 3.2.6. Retrieval

The response period was associated with the most widespread activation (see Figure 4). ERDs were observed in left DLPFC (BA 9/46), left inferior frontal gyrus (BA 44/45), left premotor area (BA 6), left superior temporal gyrus (BA 22), right inferior frontal gyrus (BA 44/45), right parietal regions (BA 40), left and right middle temporal region (BA 21) and precuneus bilaterally (BA 7) (*P* < .005 corrected). Beta ERDs peaked after the presentation of the probe (from 0 to 100 ms) in left DLPFC and bilaterally in the inferior frontal gyrus. Subsequently (from 100 to 300 ms) ERD was located over a distributed network including left DLPFC, left inferior frontal gyrus, left superior temporal gyrus, right inferior frontal gyrus, and bilaterally over precuneus areas (Figure 5).

## 4. Discussion

The aim of the current study was to investigate beta oscillatory activity during the anticipation of WM trials and the performance of the Sternberg task. Before the presentation of WM trials significant activation was detected in a network of regions that has previously been identified in several neuroimaging studies using the Sternberg task. A time frequency analysis revealed that beta activity was elicited in DLPFC during all task phases, suggesting that it may index executive processes. The findings support the hypothesis of a prefrontal modulation of higher order associative areas relevant for the upcoming task. The most interesting results, however, correspond to the remarkable difference between beta frequency modulation during preparatory periods and task epochs. While states of preparation evoked an enhancement of energy in the beta frequency, a significant decrease in beta power was observed during task related processes.

### 4.1. Oscillations in Relation to Task Performance: Encoding, Delay, and Retrieval

#### 4.1.1. Encoding

Encoding was associated with widespread beta power reduction in visual areas. The decrease of beta power in these regions may have been associated with extraction of information during presentation of the set of 5 letters. A later phase of beta power decrease become apparent in left DLPFC, and temporal regions about 1000 ms after the trial onset. The timing of this beta left lateralized ERD responses suggests that it may index aspects of the encoding processes. Similar findings have been observed at left temporal and frontal electrodes during the encoding phase of an auditory WM task [8].

#### 4.1.2. Delay

Our results highlighted an oscillatory network centered on prefrontal and visual areas during the delay, in which beta power decrease predominates in prefrontal regions, whereas an increase in beta power predominates in visual regions. Our results are at variance with others who have shown enhanced beta power in both frontal as well occipital areas during short-term maintenance of an object [31]. This inconsistency is probably due to intervening variables such as task difficulties or task complexity, which are known to have effects on the amplitude of beta power [21].

#### 4.1.3. Retrieval

During the response period beta power decreased across a distributed WM network.

 It could be argued that beta activity is related to the preparation of a response. It should be noted, however, that in order to avoid a confounding of activity related to response execution we subtracted the ERD in the control task from ERD in the WM task. Thus, the residual ERD effects in DLPFC may be attributed to the retrieval processes that include mentally scanning the items maintained in WM. This is consistent with a recent experiment that has investigated MEG activation in subjects performing a WM task [32]. Decreased beta power in DLPFC was reported bilaterally in the time window centered on the response period.

#### 4.1.4. Anticipation Activity

Several studies have examined brain activity during the preparation period of task switching [33–35] and block transition [36]. To avoid possible interference with set shifting signals (e.g., “discarding” the control task set and “installing” the working memory task set) we investigated anticipation using only the second and the third trials from each block. Since WM trials were presented blockwise, subjects knew which type of task they would be presented with on the next trial and so their attention likely was focused on the presentation of that particular type of task. Thus, an important determinant of performance in our paradigm might be the grade of specific preparation. 

It could be argued that the observed prefrontal activity reflects the maintenance of the sequence of trials in working memory and the updating of the current position in the sequence within each trial. Although we cannot rule out this possibility, there is no reason why participants should have used such expensive strategies. It is also possible that the activity in prefrontal cortex only reflects general preparatory processing. However, when stimuli are presented blockwise a subject's tonic alertness remains at a relatively stable level throughout the prestimulus interval [37]. In contrast, in the present study the brain activity preceding the task was transient rather than being sustained. The activated network during the preparatory periods included bilateral DLPFC, left inferior frontal gyrus, temporal, and parietal regions. The right DLPFC has been implicated in neural mechanisms of top-down control in a spatial attention task [38]. However, in our study the level of spatial attention required was minimal. It may be that activity in the right DLPFC reflects the DLPFC's involvement in the reorienting of attention from a resting state during the intertrial intervals, refocusing on the ongoing task [39]. 

 The association between the left DLPFC and cognitive control during the anticipation of an upcoming task has been reported previously [40, 41]. Consistent with our results, Sakai and Passingham [3] observed BOLD activity in Broca's prior to the actual trial in a verbal WM task. In the current study, left inferior frontal gyrus, temporal regions and the inferior parietal lobe also had significant activation during the preparatory intervals. Involvement of each of these regions is consistent with the well-defined neural architecture of WM as reported in numerous earlier functional imaging studies. A plausible interpretation of these findings is that the left DLPFC is involved in priming cognitive resources in areas relevant for the upcoming task in anticipation of expected WM processing. This interpretation is consistent with the functional connectivity of the left DLPFC and the role of the prefrontal cortex in cognitive control as suggested by studies in primates and humans [42]. In FMRI studies, activities in the DLPFC and the left inferior frontal gyrus have been correlated with the ability to resolve proactive interference from irrelevant information (stimuli appeared in the immediate preceding trials) in the Sternberg paradigm [43]. However, it should be noted that in the current experiment we attenuated interference across trials as there were no stimulus overlaps between the strings (or probe) that followed one another. We did not find a significant correlation between prefrontal activity and RTs. Similar findings have been reported by Sakai and Passingham [3]. This lack of a direct correlation between the physiological response during preparatory activity and the behavioral data might be due to the task's minimal variance in performance. Moreover, the variability in preparatory neural signals from trial to trial may reduce the correlation between participants' performance and predictive signals [44].

#### 4.1.5. Anticipation Activity and Task Related Processes

Comparing the preparatory and WM neural correlates we observed that left DLPFC and the inferior prefrontal gyrus showed effects of both preparatory and WM related processes during the delay and retrieval periods. Unlike those prefrontal regions, other cortical areas were activated exclusively during task performance, suggesting differences in neural preparatory periods and task performance [3]. 

The functional role of these regions can be also dissociated based on the difference in the beta amplitude we observed. Assuming that beta ERD and ERS are, respectively, correlates of increase and decrease in cortical activation [13, 14], we speculate that increases in beta activity might be considered a mechanism of “active inhibition”. This activity might set those cortical areas that are task relevant in a less excitatory state allowing the network to receive new information more efficiently. Accordingly it has been suggested that the anticipation of upcoming events might be implemented as a process that reduces the threshold levels of neurons in cortical areas [45]. A similar increase in beta power has been reported over the sensorimotor areas, the so-called “beta rebound”, within 1 s after movement execution [46]. Thus, it could be argued that the beta ERS we observed during the intertrial periods might reflect some sort of “recovery” signal. This interpretation does not, however, apply to the present results. In our study, beta power increases occurred at the end of the intertrial interval rather than following the termination of the previous trial. On the other hand localized beta energy decrease is generally considered an indicator of cortical activation reflecting small neuronal assemblies working in a relatively independent manner [13, 47]. Thus, beta power decrease associated with distinct task phases might reflect online related cognitive processes. Admittedly, this account is speculative, and other interpretations are possible. To make the results comparable with others in the literature we set the beta frequency range at 14–30 Hz. However, the structure of beta band includes discrete beta components that probably reflect the possible role of different substrates and related harmonics effects. Earlier studies [8, 25] observed beta rhythm responses during the performance of working memory tasks and reported that the beta ERD and ERS responses was narrower than the corresponding beta frequency band (14–30 Hz). In the present study, no such narrow frequency beta responses were observed. Such discrepancies between the earlier studies by Pesonen et al. and the current data may be partially explained by the different signal analysis methodologies and design. Moreover, it has been noted [25] that while the beta ERD responses were most pronounced in the posterior recording sites, the beta ERS responses were greatest in the frontal electrodes suggesting the presence of two distinct beta response systems and related harmonic effects. Indeed, it has been suggested that beta activity at occipital sites may in part represent an “alpha fast variant” related to visual processing in the same way as the posterior alpha rhythm [48]. In exploratory analyses (principal component analysis; not reported) we found suggestive evidence that frontal and occipital cortex may differ from each other in terms of beta responses was obtained from, indicating that the brain activation differs quantitatively and qualitatively across regions, such as the posterior visual regions differing from frontal regions. 

 We are aware of the limitations of the current study and of our observations. First, MEG is a noninvasive neurophysiological technique that measures the magnetic fields generated by neuronal activity of the brain. The spatial distributions of the magnetic fields are analyzed to localize the sources of the activity within the brain, and the locations of the sources are superimposed on anatomical images, such as MRI, to provide information about both the structure of the brain. MEG has a very high temporal resolution with time scales on the order of milliseconds but is limited in its spatial resolution and cannot resolve activity from cortical nearby locations as accurately as fMRI. Second, to investigate task preparation during the preparatory periods an explicit cue might have presented in advance of the task allowing preparation of an upcoming WM or control task. However, a recent event related fMRI study has shown that similar PFC network is activated in both cue-target trials as well as in no-cue target suggesting that task preparation is necessary whenever a task has to be implemented [33]. Third, to establish a direct causation of prefrontal modulation in higher order cortical regions analyses of corticocortical interaction should have been performed. Our results, however, are compatible with the top-down influence of the left prefrontal cortex during language processing and left inferior prefrontal cortex playing a critical role in verbal working memory [49]. Fourth, we investigated the beta frequency only; therefore, we cannot rule out that similar effects as in the beta frequency might be also present in other frequencies. For example, power increase in the alpha band have been associated with active inhibition [12]. Thus, it would be especially interesting investigate whether power increase in the anticipatory phase of WM tasks might also found in the alpha frequency range. The functional distinction between the online processes associated with WM trials and preparatory processes must be interpreted with caution until they are replicated by future studies using similar methodology.

## 5. Conclusion

Our results provide support for the hypothesis that, during preparatory states, the prefrontal cortex is important for biasing processing in higher order brain regions that are going to be engaged in the upcoming task. While beta ERS may provide active suppression of anticipation bringing the network back to a preactive state, ERD likely reflects the cognitive control related to online WM processes.
